# Effects of natural products on skin inflammation caused by abnormal hormones secreted by the adrenal gland

**DOI:** 10.3389/fphar.2023.1156271

**Published:** 2023-05-03

**Authors:** Wei Xie, Ce Zhang, Tian Wang, Jianshe Wang, Fenghua Fu

**Affiliations:** School of Pharmacy, Key Laboratory of Molecular Pharmacology and Drug Evaluation, Ministry of Education, Collaborative Innovation Center of Advanced Drug Delivery System and Biotech Drugs in Universities of Shandong, Yantai University, Yantai, Shandong, China

**Keywords:** natural products, skin inflammation, abnormality of hormone, hormone of adrenal gland, adrenal gland

## Abstract

The cortex of adrenal gland produces glucocorticoid, mineralocorticoid, and androgen. The medulla of adrenal gland secrets catecholamines. These hormones play an important role in regulating blood pressure, metabolism, and homeostasis of glucose or electrolytes. Hypersecretion or hyposecretion by the adrenal gland will cause a complex cascade of hormone effects and lead to diseases, including Addison’s disease, Cushing’s syndrome, and congenital adrenal cortical hyperplasia. Skin is the largest organ of body. It provides protection and acts as a barrier against external damage factors like infectious organisms, chemicals, and allergens. Endocrinologic disorders often induce cutaneous abnormalities. According to the previous evidences, natural products have the potential properties for attenuating skin disorders and improving dermatologic symptoms by inhibiting inflammation through MAPK or PI3K/AKT-dependent NF-κB pathways. The natural products may also promote skin wound healing by inhibiting the production of matrix metalloproteinase-9. We systematically searched the relevant articles from databases, including PubMed, Embase, and Cochrane library databases, to review the effects of natural products on skin disorders. This article summarized the effects of natural products on skin inflammation caused by abnormal hormone secreted by adrenal gland. And the published papers indicated that natural products might be a potential source for treating skin diseases.

## 1 Introduction

Skin is a diverse and extensive organ of the human body. The functions of the skin are important for the healthy and aesthetic conditions (Michalak, 2022). The skin is a well-known reflection of internal pathologic conditions. The unfavorable appearance resulting from cutaneous inflammation affects the mental condition of the patient. And both sides mentioned above play a key role in development and therapy of cutaneous diseases (Rashtak and Pittelkow, 2008). Immune dysregulations in allergic reactions, infections, and internal disorders usually cause cutaneous inflammation. Hormones secreted by adrenal gland play an important role in maintaining physiological function of body. Excessive or insufficient secretion of hormones will cause many diseases. These diseases may induce inflammation-mediated skin diseases. The inflammation of skin is also a main sign of endocrine disorder and chronic autoimmune inflammatory diseases, such as lupus erythematosus, atopic dermatitis, psoriasis, atopic dermatitis, and excessive pigmentation (Yang et al., 2022). Inflammation is an approach to protect cells and to regulate the stimulus including allergens, mechanical stress, irritants, toxins, and pathogens. However, excessive inflammation can lead to tissue lesion. Inflammation is a complex process which mediates skin healing and repairs after injury. Meanwhile, it is a main feature of skin disorders (Nicolaou, 2013). Inflammation is characterized by symptoms such as pain, heat, itching, redness, and swelling.

At present, the marketed drugs aim to reduce inflammation through regulating and/or suppressing immune responses and then improve function of skin, thus ameliorate clinical symptoms and signs with pruritus (Huang et al., 2022). It is well known that agents targeting IL-13, IL-4 (e.g., lebrikizumab, tralokinumab, and dupilumab) and JAK inhibitors (e.g., abrocitinib, upadacitinib, and baricitinib) are efficacious in treating atopic dermatitis. Novel topical agents to treat atopic dermatitis include phosphodiesterase 4 and JAK/STAT inhibitors (Suga and Sato, 2019; Munera-Campos and Carrascosa, 2020).

UP to date, there are no effective agents which can prevent the recurrence of skin disorder or block skin inflammation. Long-term treatment is difficult to be carried out because of the adverse reaction of therapeutic drugs and the inefficiency after application. There is a growing need to find valid therapeutic strategies to cure chronic inflammatory cutaneous diseases (Yang et al., 2022). Natural products or specifically botanicals are promising therapeutic agents in contemporary medicine. A suitable dosage of natural products or botanicals is effective to ameliorate the skin diseases and has less adverse effects when compared to most pharmaceutical medicines. It reported that curcumin can attenuate the skin disorders just causing gastrointestinal discomfort which is a mild adverse event (Zhang et al., 2022). Long-term application of escin does not induce drug resistance. And escin shows a more effective antioxidant activity, cell protection, and anti-aging properties (Dong et al., 2020). Hydroalcoholic extract of *Sapium glandulatum* is a potential source of anti-inflammatory compounds for the treatment of the inflammation-derived skin diseases. The mechanism of action may be partially related to activating the glucocorticoid receptor. Research showed multiple treatments with Hydroalcoholic extract of *S.apium glandulatum* did not cause major adverse effects when evaluated in the skin atrophy model (Mendes et al., 2016).

**FIGURE 1:**
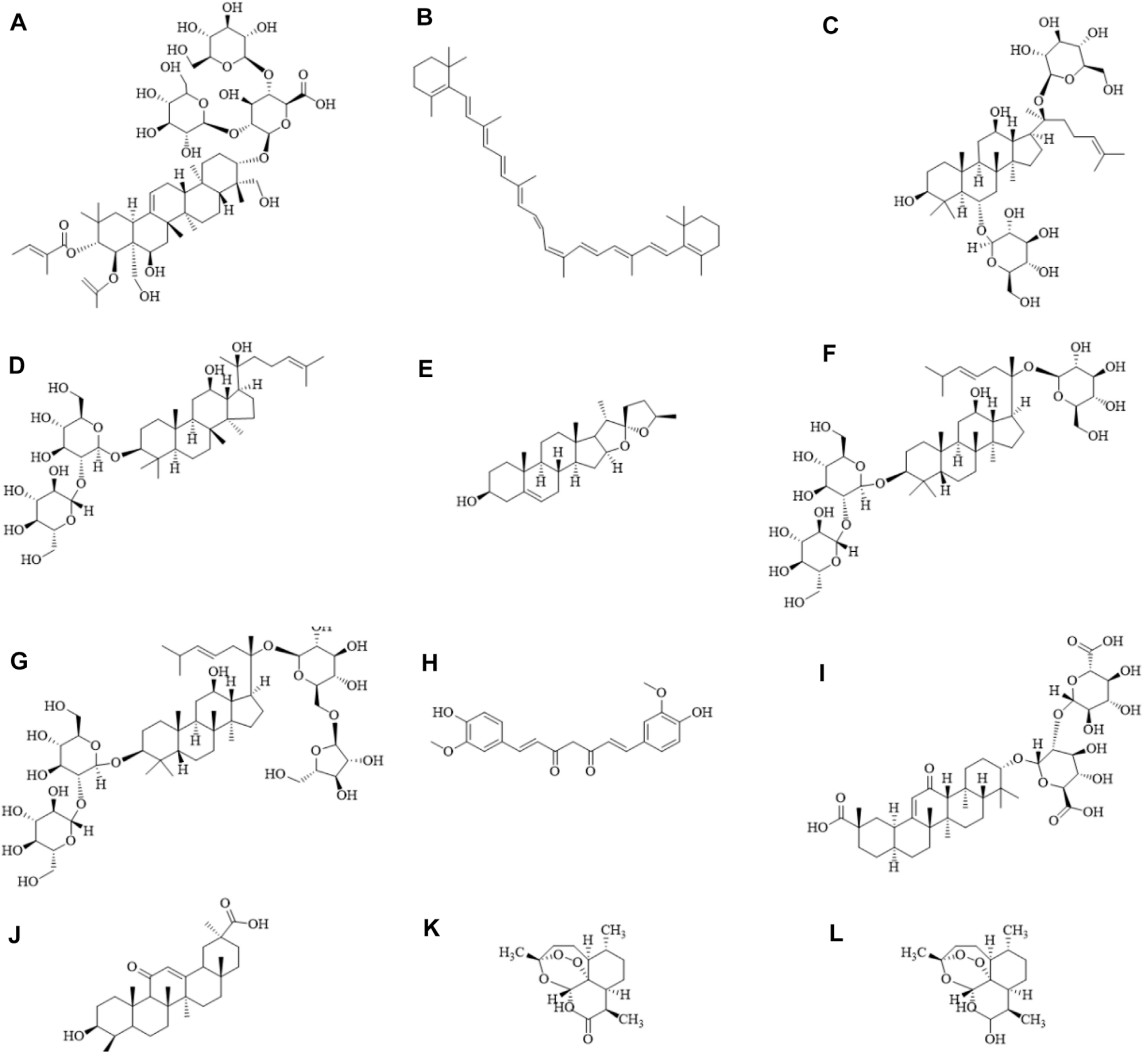
Diagram of the chemical formula structure of natural products. **(A)** Escin, **(B)** β-carotene, **(C)** Ginsenoside Rg1, **(D)** Ginsenoside Rg3, **(E)** Diosgenin, **(F)** Ginsenoside Rd, **(G)** Ginsenoside Rc, **(H)** Curcumin, **(I)** Glycyrrhizic acid, **(J)** Glycyrrhetinic acid, **(K)** Artemisinin, **(L)** Dihydroartemisinin. The schematic diagram was drawn using ChemDraw 20.0 software.

Escin (Figure 1A), an extract of *Aesculus chinensis* Bge., is a compound showing an anti-inflammatory effect. The compounds in *A.esculus chinensis* Bge. also have many beneficial effects on the skin and therefore they are used in cosmetic skin-care products (Sarikurkcu et al., 2020). Previous study showed that escin could attenuate the symptoms of atopic skin inflammation (Jeong et al., 2018). In diabetic rats, escin improved wound healing through its antioxidant activities and anti-inflammatory effects (Zhang et al., 2015). Furthermore, it reported that β-carotene (Figure 1B) which is abundant in fruits and vegetables has protective effects against skin injury induced by exposure of UV light (Balić and Mokos, 2019). β-carotene is also used to treat erythropoietic protoporphyria. It reports that β-carotene inhibits inflammation of skin by suppressing the expression of inflammatory factors, promoting expression offilaggrin in atopic dermatitis-like skin, as well as reducing the activity of matrix metalloproteinases (Kake et al., 2019; Takahashi et al., 2019). Ginsenosides extracted from Panax ginseng C. A. Mey (ginseng) are found to inhibit skin inflammation. It is reported that ginsenoside Rg1 (Figure 1C) could attenuate BB-UVB-induced resistance of glucocorticoids in keratinocytes trough Nrf2/HDAC2 signaling pathway (Li et al., 2016). Ginsenoside Rg1 abolishes imiquimod-induced psoriasiform dermatitis by lowering psoriasis severity index score and damage area, skin thickness, lipid peroxidation, and inflammation through downregulating NF-κB signaling pathway (Shi et al., 2019). Ginsenoside Rg3 (Figure 1D) ameliorates cutaneous disorders by blocking MDM2/HIF1α signal pathway (Han et al., 2021).

Recently, a large quantity of researches focused on using herbal natural products as potential agents to inhibit the skin inflammation. In this article, we review the anti-skin inflammatory effects of natural products, including diosgenin, ginsenoside, curcumin, glycyrrhizic acid, glycyrrhetinic acid, artemisinin, and dihydroartemisinin. The contents mainly focused on the skin inflammation caused by abnormal hormone secretion of adrenal gland.

## 2 Physiological function of hormones secreted by the adrenal gland

Adrenal gland is composed of adrenal medulla and adrenal cortex. Based on the physiological functions, adrenocortical hormones are divided into three categories: glucocorticoid, mineralocorticoid (aldosterone), and sex hormones. Adrenocorticotropin (ACTH) is secreted by the pituitary gland. ACTH binds to its receptor and promotes the conversion of cholesterol from cytoplasm to mitochondria into pregnenolone. Then, with the action of a series of enzymes, adrenocortical hormones are produced, which also become steroids hormone. Adrenal medulla secreted hormones including epinephrine, norepinephrine, and dopamine. The hormones secreted by adrenal glands are crucial for homeostasis of sodium, homeostasis of glucose, regulation of blood pressure and metabolism. Secretion of too much or not enough of the hormone will be life-threatening. Understanding the biological functions of adrenal hormones is a precondition to the treatment of adrenal gland disease (Kanczkowski et al., 2017).

**FIGURE 2:**
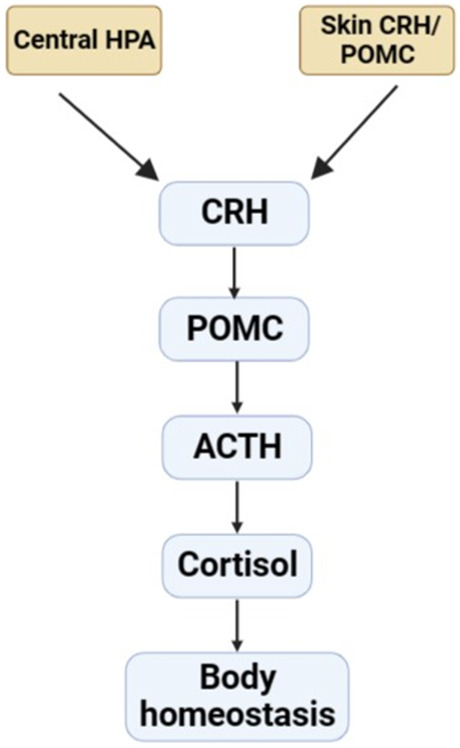
CRH/POMC skin system.

In addition, sebaceous gland of skin exhibits an independent peripheral endocrine function. Previous studies reported there was the presence of a corticotropin-releasing hormone (CRH) system in human sebaceous cell *in vitro* (Deacon et al., 2000; Krause et al., 2007). The mRNA and protein of CRH and the receptors are found at the skin of rodent or human. Skin also expresses pro-opiomelanocortin (POMC) and its products, including ACTH. It also demonstrates that POMC mRNA and protein are expressed in the skin of mouse during anagen, but not in telogen (the resting phase). And the immunolocalization of the POMC product is restricted to the sebaceous gland (Rassouli et al., 2018). Recent studies have shown that skin cell contain the apparatus which are necessary for production of glucocorticoid (Slominski et al., 2020), androgens (Ceruti et al., 2018; Bienenfeld et al., 2019), and estrogens (Zouboulis et al., 2022). Meanwhile, these hormones were transformed from precursors or cholesterol (Slominski et al., 2013). Furthermore, the CRH/POMC skin system fulfils analogous functions to the hypothalamic-pituitary-adrenal stress (HPA) that may act as a cutaneous defense system (Figure 2). Human skin expresses elements of the HPA axis including POMC, CRH, the CRH receptor-1 (CRH-R1), enzymes of corticosteroid synthesis and then synthesizes glucocorticoids. In melanocytes and fibroblasts, CRH-induced CRH-R1 stimulation upregulates the expression of POMC and the production of ACTH through activating cAMP dependent pathway (Slominski et al., 2013). It can act as a coordinator and executor of local responses to stress, preserving body homeostasis (Ziegler et al., 2007).

## 3 Effect of hormones secreted by the adrenal gland on skin inflammation

The endocrine system interacts with integumentary systems through a cohort of mechanisms. In the endocrinopathies, the dysregulation of endocrine hormones will lead to dermatologic disease (Lause et al., 2017). Abnormal hormones secreted by adrenal gland can also cause skin inflammation, and this is an important pathogenesis of skin diseases.

Glucocorticoids are cardinal regulators of epidermal growth, differentiation, and homeostasis (Bigas et al., 2018). Glucocorticoids are also widely used in pharmaceutical field. Biological activities were detected with topical glucocorticoids which make them meaningful in cutaneous medicine and diseases of the skin. Glucocorticoids have antiproliferative, anti-inflammatory, immune suppressive, and vasoconstrictive properties in treating cutaneous disorders (Niculet et al., 2020). However, the usage of glucocorticoids by a topical or inhaled route (local or systemic) is associated with lots of adverse side effects (Schoepe et al., 2006; Guillot, 2013). Metabolic side effects are characterized by skin atrophy (Jung et al., 2017), superficial erosions of skin, easy bleeding of skin, and thin skin. Other adverse side effects include the delayed wound healing, striae, hypertrichosis, and acne of papulopustular type (Guillot, 2013). Glucocorticoids can result in immunosuppressive side effect and lead to infection (McDonough et al., 2008), such as parasites diseases (Norwegian scabies), fungal dermopathy (dermatophytosis), viral infections, or bacterial infections. Aldosterone is recognized for its action on the kidney and the cardiovascular system. It modulates deposition of extracellular matrix in skin (Mitts et al., 2010). Human cutaneous tissues can express the local renin-angiotensin-aldosterone system (RAAS). The RAAS in skin exerts a moderating function in aging, cutaneous heating adaptation, wound healing, scarring, and epidermal proliferation. There are also signs demonstrating its role in the regulation of sebum secretion and hair growth. Impaired cutaneous wound healing relating with scleroderma, psoriasis, cancer development, and diabetes may be associated with changes in cutaneous RAAS (Aleksiejczuk et al., 2019). Adrenal gland produces a large quantity of androgens, such as dehydroepiandrosterone. Cutaneous tissue is a major target of androgens. Androgen receptors are expressed in the hair, sebaceous glands, epidermis, and dermis. Dysfunction of androgens in the adrenal gland and/or the skin is associated with acne (Horwitz et al., 2019), hirsutism (Fuchs et al., 2019), and androgenic alopecia (Nestor et al., 2021). The diseases of the adrenal cortex can be viewed from the dual perspectives of hyperfunction and hypofunction. Excessive corticosteroids may lead to several disorders including the adrenogenital syndrome, primary hyperaldosteronism, and Cushing’s syndrome. Addison’s disease and selective hypoaldosteronism are caused by adrenocortical hormone deficiency (Lause et al., 2017). It reported that Cushing’s syndrome can cause acanthosis nigricans, skin atrophy, bruising, hyperpigmentation, and steroid acne (Erden et al., 2019). While the cutaneous manifestations of Addison’s disease include oral mucosa, genital skin, recent scars, vermilion border, frictional surfaces, darkening of the skin in sun-exposed areas and hyperpigmentation of the palmar creases (Nieman and Chanco Turner, 2006). Dysregulation of androgens in the skin and/or the adrenals is associated with androgenic alopecia, hirsutism, and acne.

In patients with low blood pressure, norepinephrine is commonly used to improve hemodynamic stability. It is reported that noradrenaline may result in peripheral gangrene (Jung et al., 2018). Noradrenaline is regarded as a life-saving medicine. Noradrenaline administration may cause an insufficiency of circulation in limbs and therefore lead to necrosis or dry gangrene in skin (Wilson et al., 2021).

The hormones secreted by the adrenal gland play a key role in maintaining body homeostasis. If adrenal gland secreted hormones too much or too little, which will cause skin inflammation leading to skin diseases.

Recent evidence suggested that responses for endocrine stress not only are under control of the central nervous system but also occur in peripheral tissue (Lian et al., 2014; Zitkovsky et al., 2021; Churilov and Milton, 2022). Skin is a target of the POMC-derived neuropeptides alpha-melanocyte stimulating hormone, beta-endorphin, or ACTH. Skin expresses POMC or POMC/CRH peptides. The levels of expression of POMC or POMC/CRH peptides are not static. It is determined by physiological changes associated with hair cycle, ultraviolet radiation exposure, immune cytokine release, or the presence of cutaneous pathology (Bocheva et al., 2019; Ocampo-Garza et al., 2020). Previous work shows that the level of CRH is increased in acne-involved skin, especially in the sebaceous glands, possibly activating pathways which affect immune and inflammatory processes leading to the development and stress-induced exacerbation of acne (Ganceviciene et al., 2009; Khamdan et al., 2022).

## 4 Effect of natural products on hormones secreted by the adrenal gland

The hormones secreted by adrenal gland play an important role in the metabolism (Sevilla et al., 2013), regulation of blood pressure (Reincke et al., 2021), and homeostasis of sodium or glucose (Kuo et al., 2015; Christa et al., 2022). Natural products may influence secretion of hormones from adrenal gland.

### 4.1 Diosgenin

Diosgenin (Figure 1E) is a natural steroidal sapogenin (Schwarz et al., 2022). Diosgenin is found in *Dioscorea alata* L., *Smilax china* L., and *Trigonella foenum-graecum* L. (Jesus et al., 2016). Diosgenin can also be extracted from roots of dioscoreae rhizoma. The extraction business is originated in Mexico. The extraction method of the steroids field was introduced to Chinese companies (Herráiz, 2017). Diosgenin is a precursor of steroid hormones. And it shares a similar steroidal structure with glucocorticoids (Junchao et al., 2017). Diosgenin has a variety of biological activities including anti-inflammatory effect (Ran et al., 2020). Diosgenin in *Dioscorea nipponica* exerts an anti-trachea inflammatory effect through suppressing the secretion of TNF-α, IL-1β, and IL-6 by the interactions with glucocorticoid receptor α (Junchao et al., 2017). Long-term usage of glucocorticoids may cause side effects and thereby limits their use. In the process of searching for compounds that are as effective as glucocorticoids but with less side effects, it is proved that diosgenin can interact with glucocorticoid receptor (GR) (Morsy et al., 2019) using computer molecular docking. The structure of diosgenin is similar as that of glucocorticoid. And diosgenin can interact with GR and then exerts its therapeutic role. It is speculated that diosgenin may replace glucocorticoid and combine with GR to regulate the secretion of glucocorticoid in adrenal gland.

### 4.2 Ginsenoside

Panax ginseng C. A. Mey (ginseng) is a traditional Chinese medicine (Liu et al., 2020). In China, it has been used to treat a series of diseases for more than 1,000 years. Ginseng is now used in the therapy of inflammation as alternative medicines. Ginsenosides, the main active ingredients of ginseng, are triterpenoid saponins that have a rigid steroidal skeleton with sugar moieties. Ginsenosides have multifarious pharmacological effects (Mancuso and Santangelo, 2017). Based on the chemical structure, ginsenosides are divided into two groups: protopanaxatriols and protopanaxadiols (Hou et al., 2021). The protopanaxadiols include ginsenoside Rb1, Rb2, Rb3, Rc, Rd, Rg3, Rh2. While the sugar moieties in the protopanaxatriols group are attached at the 6-position of ginsenosides including Rf, Re, Rg1, and so on. Ginsenosides show different pharmacological effects and the mechanisms of actions are due to their different chemical structures. Ginsenosides have similar structures to steroids (Yue et al., 2007) and they can prevent stress-associated diseases through reducing glucocorticoid secretion (Zhang et al., 2016). Studies suggested that ginsenoside Rd (Figure 1F) attenuated corticosterone secretion induced by ACTH *via* inhibiting the MC2R-cAMP/PKA/CREB signal pathway in adrenocortical cells of mouse (Jin et al., 2020). It has also been reported that ginsenoside Rg1 displayed an antidepressant activity *via* regulating hypothalamic-pituitary-adrenal axis to decrease serum corticosterone level (Mou et al., 2017). The effects of preparations of saponin mixture and ginsenosides on plasma corticosterone and corticotropin in rats were detected *via* the competitive protein binding methods and radioimmunoassay. Ginseng saponin was found to act on the hypophysis primarily and/or hypothalamus. And it stimulated ACTH secretion, resulting in increased biological synthesis of corticosterone in the adrenal cortex. (Li et al., 2014). In addition, the total saponin of ginseng and ginsenoside Rc (Figure 1G) significantly attenuated the ACTH-induced increase of plasma corticosterone (Kim et al., 2003). These results demonstrated that ginsenosides which has different structures and act as analogues of steroids can regulate the hormone secretion of adrenal gland.

## 5 Treatment of natural products on skin inflammation caused by abnormal hormones secreted by the adrenal gland

### 5.1 Curcumin

Curcumin (Figure 1H) is a compound which is extracted from *Curcuma longa* L. (turmeric) (Farzaei et al., 2018). Turmeric has various pharmacological activities and curcumin is the major active component in turmeric (Kocaadam and Şanlier, 2017; Chopra et al., 2021). Previous studies indicated that curcumin could inhibit vascular remodeling. Curcumin shows antitumor, antioxidant, and anti-inflammatory properties (Aggarwal and Harikumar, 2009; Walker and Mittal, 2020; Mahjoob and Stochaj, 2021). Hormone abnormalities of adrenal gland are the major cause leading to Cushing’s syndrome (Staack et al., 2020; Uehara et al., 2020), Addison’s disease (Jabbour, 2003; Nieman and Chanco Turner, 2006). Hormone abnormalities of adrenal gland can also induce skin inflammation including acne (Nikolakis et al., 2016), skin atrophy (Schoepe et al., 2006), bruising (Niculet et al., 2020), hyperpigmentation (Benner et al., 2019), hirsutism (Bienenfeld et al., 2019), androgenetic alopecia (Alesci and Bornstein, 2000). Up to date, there are a growing number of evidences suggesting curcumin can be used to improve chronic pain, inflammatory dermatoses, skin infections, psoriasis (Panahi et al., 2019), acne, skin cancer, as well as dyspigmentation (Nguyen and Friedman, 2013). It is reported that hairless were topically treated with 500 µL of solutions containing 10% curcumin, 3% ginger extract or the combination of the two, daily for 21 days, ameliorated abrasion wound healing in the corticosteroid-impaired skin in rats. In the healed skin, the production of collagen was increased while matrix metalloproteinase-9 was decreased (Bhagavathula et al., 2009). Curcumin can improve psoriatic cutaneous lesions alone or in combination with other medicines *via* inhibiting the release of inflammatory factors and keratinocyte proliferation (Zhang et al., 2022). Curcumin oral administration (40 mg/kg, for 20 days) resulted in significant reduction of the serum levels of IL-2, IL-12, IL-22, IL-23, INF-γ, and TNF-α in psoriatic mice, reducing psoriasis-associated inflammation as well as hyper-proliferation of keratinocytes. Clinically, a phase II clinical trial confirmed the efficacy of oral curcumin on cutaneous symptoms of plaque psoriasis, reporting an excellent safety profile (Vollono et al., 2019). A clinical study investigated the use of an herbal combination gel containing turmeric, rosemary, and gotu kola (Tricutan^®^) for improving signs of photoaging. The treatment showed a significant improvement in skin firmness and self-evaluations after 4 weeks usage (Sommerfeld, 2007). Clinical applications of curcumin are difficult because of its insufficient solubility, chemical instability, and poor absorption. Recent reports demonstrated that these limitations can be overcome by using a nanotechnology-based delivery system.

### 5.2 Glycyrrhizic acid and glycyrrhetinic acid

Glycyrrhizae Radix et Rhizoma is a native natural products to Asia and Mediterranean area (Sabbadin et al., 2019). Glycyrrhizae Radix et Rhizoma contains a variety of active constituents. It has been used as medicinal agent since ancient times. Glycyrrhizae Radix et Rhizoma shows many biological activities including anti-inflammatory effect (Yang et al., 2017), neuro-protective effect (Lim et al., 2018), anti-cancer action (Wen et al., 2021), and hepatoprotective activities (Kuang et al., 2017). The fresh leaves of Glycyrrhizae Radix et Rhizoma are traditionally used for wounds (Liu et al., 2018). The roots of licorice have been used as a traditional drug to treat cough and stomachache (Liu et al., 2018). Moreover, the stem of Glycyrrhizae Radix et Rhizoma was used to control the symptom of diabetes (Gaur et al., 2014). Glycyrrhizic acid (Figure 1I) is one of the main components of Glycyrrhizae Radix et Rhizoma (Liu et al., 2018). It has been confirmed that glycyrrhizic acid is responsible for the main bioactivities of Glycyrrhizae Radix et Rhizoma’s, such as anti-inflammatory activity. Both ears of the mice were topically treated with a total of 30 μL of Glycyrrhizic acid solution at doses of 50 mM, 100 mM, 150 mM, and 200 mM at 15 min before topical application of a total of 30 μL of TPA solution (25 μg/mL). It demonstrated that glycyrrhizic acid inhibited inflammatory responses *via* blocking PI3K/Akt signaling pathway, thereby attenuating NF-κB activation in skin inflammation. Glycyrrhizic acid can downregulate the expression of inducible nitric oxide synthase and cyclooxygenase-2 (Liu et al., 2018). Previous data suggested that the deficiency of glucocorticoid in psoriasis skin induced a localized and sustained inflammatory response (Sarkar et al., 2017). An intraperitoneal injection with glycyrrhizic acid at dose of 50 mg/kg/d for 7 days can attenuate dermatitis and improve psoriasis-like cutaneous inflammation in mice through reducing intercellular adhesion molecule-1 expression. Glycyrrhizic acid regulates the ERK/p38 MAPK and NF-κB signaling pathways in keratinocytes (Xiong et al., 2015). Patients of erythrodermic psoriasis with bullous pemphigoid were treated with methotrexate at a dose of 15 mg weekly and compound glycyrrhizin (150 mg/d). After 2 weeks, the patient’s condition had been improved. And the diffuse flushing and the infiltrative swelling were ameliorated (Si et al., 2014). Clinical study showed that, glycyrrhizin and UVB combination therapy led to improvement active-stage generalized vitiligo in disease stage from active to stable (Mou et al., 2016). Glycyrrhizic acid being hydrolyzed and then transformed into glycyrrhetinic acid (Figure 1J) in stomach and duodenum (Armanini et al., 2004), which is responsible for its pharmacological properties. 18β-glycyrrhetinic acid is a major active component of Glycyrrhizae Radix et Rhizoma root and has a wide range of dermatological applications. From the viewpoint of dermatology and cosmetology, inflammation process is the basis of many skin disorders, such as ageing skin, atopic skin, or acne. 18β-glycyrrhetinic acid exerts an anti-inflammatory activity through inhibiting the expression of pro-inflammatory genes, suppressing the production of inflammatory cytokines, and blocking the transformation of arachidonic acid into pro-inflammatory leukotrienes (Kowalska and Kalinowska-Lis, 2019). 18β-glycyrrhetinic acid, a 18β-glycyrrhetinic acid-3-O-β-D-glucuronid metabolite in human intestine, also reduced skin inflammation and the passive skin anaphylaxis (Park et al., 2004). It is reported that a dose of 50 mg/cm^2^ 18β-glycyrrhetinic acid cream or vehicle cream (without 18β-glycyrrhetinic acid) was applied twice daily for 7 consecutive days alleviates psoriasis-like diseases in mice and induces apoptosis of keratinocytes *via* PI3K/Akt signaling pathway (Gao et al., 2020). All patients of scalp seborrheic dermatitis were instructed to rinse and massage their scalps with 6% glycyrrhetinic acid. After 10–20 s, rinsing off with water, followed by a second massage for 1–2 min with the same product, every day for 5 weeks. The results found that this new formulation can attenuate seborrheic dermatitis of the scalp (Wang et al., 2022). In clinic, glycyrrhizic acid or glycyrrhetinic acid alone were not effective. Therefore, they are often combined with other drugs to elevate efficacy.

### 5.3 Artemisinin and dihydroartemisinin

Artemisinin (Figure 1K) is a sesquiterpene lactone isolated from artemisiae annuae herba which is a Chinese plant. It has been used for treating different forms of malarial parasites (Uehara et al., 2020). The potential application of artemisinin and its derivatives in coronavirus disease 2019 (COVID-19) treatment has also been recently proposed (Shi et al., 2022). Studies also demonstrate that artemisinin possesses effects of anti-inflammation (Wang et al., 2017) and anti-angiogenesis (Wei and Liu, 2017). Rosacea is a skin disorder showing vascular and immune system dysfunction. Disturbance of corticosteroid homeostasis (Nikolakis et al., 2016; Saric-Bosanac et al., 2020) and topical glucocorticoids treatment (Hengge et al., 2006; Nikolakis and Zouboulis, 2014) play a role in rosacea. In a mouse model, artemisinin was diluted in filtered DMSO and 200 mg/kg feed by means of gavage daily for consecutive 7 days. It found that artemisinin ameliorated rosacea-like dermatitis *via* inhibiting IL-1β, IL-6, and TNF-α. Artemisinin also reduces the productions of chemokines of immune cells, such as CXCL2, CCL2, CCL20 and CXCL10 (Yuan et al., 2019). Dihydroartemisinin (Figure 1L) is an active metabolite of artemisinin (Dai et al., 2021). It has been used for the treating malaria and show a beneficial efficacy. Dihydroartemisinin showed good bioavailability and anti-malarial effects (Morris et al., 2011; Dai et al., 2021). Recent studies confirmed that dihydroartemisinin inhibited melanoma through regulating immunity and cell apoptosis (Yu et al., 2020). Dihydroartemisinin also alleviates atopic dermatitis by inhibiting mast cell infiltration (Xue et al., 2020). Studies demonstrate that the mice in DHA-treated groups were intraperitoneally administered with dihydroartemisininat at dose of 25 or 50 mg/kg/day for 7 days, which can attenuate psoriasis through inhibiting memory CD8^+^ T-cells (Chen et al., 2020), targeting fibroblast growth factor receptor 1 to IL-17A (Chen et al., 2022), and regulating the IL-23/Th17 axis (Liu et al., 2021). Further randomized, comparative clinical trials are needed in order to clarify the potential role of dihydroartemisinin in the treatment of skin inflammation. Nevertheless, the clinical application of artemisinin is limited due to its poor solubility and short plasma half-life. However, the problems mentioned above can be solved by preparing controlled release formulations.

Other natural products also alleviate skin inflammation induced by abnormal hormone secretion of adrenal gland through a range of mechanisms (Table 1). For example, quercetin improved skin wound healing by increasing the expression of growth factors *via* Wnt/β-catenin signaling pathway, by inhibiting inflammation, or by enhancing the migration and proliferation of fibroblasts (Mi et al., 2022). Aloesin ameliorates inflammation through regulating Smad and MAPK/Rho signaling pathways. These findings indicate that aloesin has a therapeutic potential for treating cutaneous wounds (Wahedi et al., 2017).

**TABLE 1:** Natural products for the treatment skin inflammation caused abnormal hormone secreted by adrenal gland.

Origin	Main components	*In vitro*		*In vivo*		Mechanism
		Cell	Dosage	Animal	Dosage and administration route	
Selaginella tamariscina	Amentoflavone (Li et al., 2016)	HaCaT cells	20 μg/mL for 2 h and then exposed to M5 for 2 days	Male BALB/c mice	25 and 50 mg/kg for 2, 4.7 days, i.g	Suppression of NF-κB
Passionflower, honey, and propolis	Chrysin (Li, Wu et al., 2020)	Keratinocytes	3, 10, or 30 μM for 24 h	Male BALB/c mice	30 mM for 6 days, ad us. ext	Reducing TNF-α-, IL-17A-, and IL-22-induced CCL20 and antimicrobial peptide release
Indigo Naturalis	Indirubin (Xie, Di et al., 2018)	γδT cells	2 μM	Male BALB/c mice	12.5, 25, 50 mg/kg/d for 7 days, i.g	Inhibiting γδT cell-mediated inflammatory responses involving IL-17 secretion and Jak3/Stat3 activation
Watermelon, pink guava, and tomato	Lycopene (Shih, Hsieh et al., 2020)	HaCaT cells	1, 5, 10 μΜ for 24 h	Male C57BL/6 mice	0.06 and 0.12 mg/mL for 5 weeks, ad us.ext	Inhibiting ICAM-1 production
Harpagophytu-m procumbens	Leucosceptoside A (Koycheva, Mihaylova et al., 2021)	HaCaT cells	20, 40, 100 μg/mL for 6 h and 24 h	-	-	Suppression of the PI3K/AKT Signaling Pathway
Aloe	Aloin (Jeong et al., 2018)	HaCaT cells	1, 5, or 10 Μm for 24 h	SKH-1 hairless mice	0.1% and 0.5% for 3 and 7 days	Modulating MAPK/Rho and Smad signaling pathways
Cruciferous vegetables	Sulforaphane (Jung et al., 2017)	C2C12 myoblasts	1, 5, 10 μM for 24 h	-	-	Regulation of the Akt/Foxo1 axis
Pigmented fruits and vegetables	Fisetin (Chamcheu, Esnault et al., 2019)	NHEKs	10–20 µM for 48 h, 1–120 µM for 24 h	-	-	Inhibiting the PI3K/Akt/mTOR and MAPK Pathways
Myrica rubra	Myricanol (Shen, Liao et al., 2019)	C2C12 cells 2.5, 5 and 10 μM for 24 h	Male C57BL/6mice	5 and 50 mg/kg for 7 days, i.p		Via a sirtuin1-dependent mechanism
Cinnamomum lauraceae	Cinnamaldehyde (Ding, Liu et al., 2021)	NHEKs	5 and 10 for 24 h	-	-	Inhibition of NF-κB and JNK signaling pathways
Paeoniae radix rubra and paeoniae radix alba	Paeoniflorin (Zhao, Di et al., 2016)	Th17 cells	2, 20 and 200 μg/mL	BALB/c and C57BL/6 mice	120 and 240 mg/kg/d for 8 days, i.g	Regulating Th17 cell response and cytokine secretion *via* phosphorylation of Stat3
Oxytropis falcata Bunge	Quercetin (Mi, Zhong et al., 2022)	HSF, MSF, L929, and HaCat cells	0–100 μM	Male C57BL/6 mice	1.5, 3 and 6 mg/mL for 2, 5 and 8days, ad us.ext	Through Wnt/β-catenin signaling pathway
Methanolic extract of root of *Asparagus racemosus*	*Asparagus racemosus* (Krishnamurthy, Garabadu et al., 2013)	-	-	Charles Foster strain albino rats	50, 100, 200 mg/kg/d for 7 days, i.g	Diminishing the HPA axis response
Water VBF extract	Vaccinium bracteatum Thunb (VBF) (Kim et al., 2003)	SH-SY5Y Cells	1, 3, 10, and 30 μg/mL	Male ICR mice	100 and 200 mg/kg/day, p.o	Regulated the ERKs/Akt signaling pathway and mediated by the regulation of monoaminergic systems and glucocorticoids
Sutherlandioside B	Sutherlandia frutescens (Sergeant, Africander et al., 2017)	COS-1 cells	10 and 30 µM	-	-	Linking anti-stress, anti-inflammatory to inhibition of steroidogenic enzymes and modulation of adrenal hormone biosynthesis
Withanolide A	Withania somnifera root (Kour, Pandey et al., 2009)	-	-	Male Swiss albino mice	0.25, 0.5, 1 and 2 mg/kg, p.o	Antistress and regulated endocrine
Ginsenoside Rb1	Panax species (Kang, Hong et al., 2019)	-	-	Male Sprague Dawley rats	40 mg/kg, i.p	Increases the plasma levels of CORT

## 6 Conclusion

**FIGURE 3:**
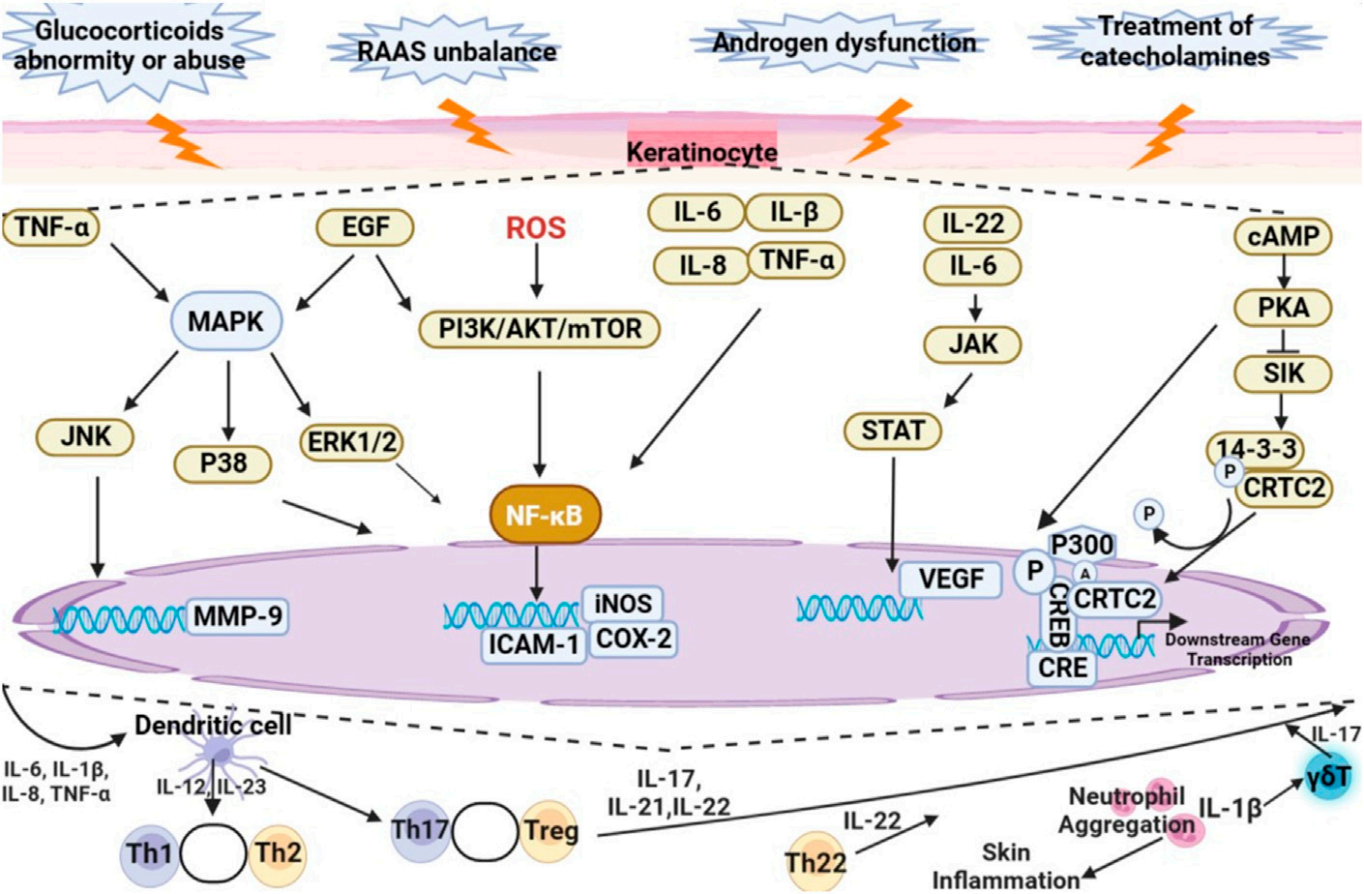
The mechanism of skin inflammation.

Disorder of hormone secretion by adrenal gland are closely associated with cutaneous lesions. Clinical presentation of skin atrophy, alopecia, psoriasis, skin pigmentation, rosacea, acne, atopic dermatitis, and hirsutism are frequently characterized by skin inflammation. The mechanism of skin inflammation is shown in Figure 3. Abnormal secretion or improper use of glucocorticoids, unbalance of RAAS, androgen secretion dysfunction and treatment of catecholamines will regulate the expression of intracellular inflammatory factors through regulating MAPK, PI3K/AKT/mTOR, JAK/STAT, and cAMP/PKA signaling pathways, resulting in skin inflammation. In conclusion, this review summarizes natural products can attenuate skin inflammation by improving the abnormality of hormones of adrenal gland, including inhibiting the proliferation of keratinocytes, increasing the production of collagen, and reducing the activity of matrix metalloproteinase-9. Based on the published-papers, it is showed that curcumin, glycyrrhizic acid, glycyrrhetinic acid**,** artemisinin, dihydroartemisinin are the main natural products to be evaluated during trials as a source of drug discovery for skin inflammation caused by abnormal secretion of hormones of adrenal gland.
